# Editorial: Molecular Biomarkers in Animal Reproduction

**DOI:** 10.3389/fvets.2021.802187

**Published:** 2021-12-01

**Authors:** Cristina Alicia Martínez, Jordi Roca, Isabel Barranco

**Affiliations:** ^1^Department of Biomedical and Clinical Sciences, Faculty of Medicine and Health Sciences, Institutionen för biomedicinska och kliniska vetenskaper (BKV), BKH/Obstetrics and Gynaecology, Linköping University, Linköping, Sweden; ^2^Department of Medicine and Animal Surgery, Faculty of Veterinary Medicine, International Excellence Campus for Higher Education and Research “Campus Mare Nostrum”, University of Murcia, Murcia, Spain; ^3^Department of Veterinary Medical Sciences, University of Bologna, Bologna, Italy

**Keywords:** omics, biomarker, reproduction, fertility, livestock species

Improving the reproductive efficiency of livestock species remains challenging the scientific community. Emerging 'omics technologies, such as genomics, transcriptomics, proteomics, and metabolomics, are helping to overcome this challenge (1, 2). These high-throughput technologies make it possible to identify the set of molecules and regulatory networks which are directly or indirectly involved in reproductive processes, and to recognize molecules that play a key role in major reproductive events (3). In this context, one of the purposes of this special issue was to highlight the increasing applicability of 'omics to identify key molecules involved in modulating main reproductive events of animal species of economic interest. Seven out of nine research papers included in this special issue used 'omics-tools, five of them using transcriptomics and the other two using metabolomics and proteomics.

It was not surprising that five out of seven papers involved transcriptomics to study reproductive processes. A large body of research is currently aimed to unravel the transcriptome of gametes and embryos of livestock species, to both explain the roles of RNAs in regulating gene pathways and to unveil RNA markers of key functional events (4). Two of these five transcriptomics-based studies explored the sperm transcriptome for two completely different purposes, one addressing the analytical procedure and the other the practical application. The research conducted by Lian et al. focused on the performance of RNA sequencing (RNA-Seq) in spermatozoa, a method that uses deep sequencing technologies to profile and quantify cellular RNAs (5). Specifically, the research evaluated the impact of the density gradient centrifugation cell purification step recommended in the protocol on the transcriptome profile of pig spermatozoa. Results demonstrated that the purification step influenced RNA abundance in a small number of genes related to translation, transcription, and metabolic processes which, associated to the purification step for removal of expected non-sperm cells as well as immature and non-functional spermatozoa, showing it compromises the soundness of the results. The research conducted by Turri et al. focused on evaluating the value of sperm micro-RNA (miRNA) profiling in predicting the fertile ability of bulls used in artificial insemination (AI) programs. They concluded that integrating sperm miRNA profiling into the battery of advanced sperm functional assessments improved our predictive capacity. The other three transcriptomic-based papers investigated female functional reproductive events. The research conducted by Pan et al. focused on decoding the complete transcriptome of granulosa cells of Chinese Buffalo ovarian follicles. Granulosa cells play a key role in the regulation of oocyte maturation and follicular atresia, the latter being one of the main factors limiting female reproductive performance in livestock species, yet involving unclear molecular mechanisms (6). The results highlighted several messenger RNAs (mRNAs), miRNAs and long non-coding RNAs (lncRNAs) with regulatory roles that were differentially expressed between granulosa cells of healthy and atretic follicles, possibly involved in critical developmental and atresia events of buffalo ovarian follicles. Del Rio and Madan explored the existence of miRNAs in media used during *in vitro* culture of bovine preimplantation embryos and identified a total of 111 miRNAs, which would regulate genes involved in early bovine embryo development. The latest transcriptomic-based work was performed by Hebbar et al. also in female buffaloes. They explored circulating miRNAs in urine to identify potential non-invasive biomarkers of estrus and highlighted the miRNA bta-mir-99a-5p, which was less abundant during estrus than during diestrus.

The other two omics-based studies used metabolomics and proteomics approaches. Metabolomics is the newest of the 'omics used to search for molecular markers of reproductive (dys)functionality displaying some advantages over the other 'omics, such as faster throughput and the ability to measure the end products of regulatory processes (7). Using a liquid chromatography-mass spectrometry-based non-targeted metabolomic approach, Mo et al. decoded the metabolic profile of atretic ovarian follicles in sows and identified 18 metabolites involved in lipid and amino acid metabolism presumably involved in follicular atresia. The last 'omic study was conducted by Fernández-Hernández et al. decoding the proteome profile of equine oviductal fluid collected before and after ovulation attempting identification of fertilization-related proteins. They identified a total of 1,173 proteins, 691 of which were included in the *Equus caballus* taxonomy and, using an iTRAQ approach, highlighted several proteins that were differentially expressed between pre- and post-ovulatory stages, some of them having a clear role in sperm-oviduct interactions and fertilization.

The special issue was also open to research or review papers exploring molecules with potential predictive value for reproductive events in livestock species and the remaining three articles served this purpose. The research conducted by Gautam et al. studied the anti-Müllerian hormone (AMH), a glycoprotein member of the transforming growth factor beta family involved in ovarian functionality (8) and considered as a reliable marker of reproductive output in dairy cattle breeds (9). The research sequenced the entire coding region of the AMH gene in water buffalo and goat females and suggested that the AMH gene has a similar and critical physiological reproductive function in both species. The research conducted by Ribas-Maynou et al. focused on sperm nuclear DNA. Early identification and removal of male breeders showing high percentages of ejaculated sperm with damaged nuclear DNA is a priority for swine AI centers to improve the fertility efficiency of AI programs (10). Despite this conclusive evidence, there is still no standardized measurement method for practical use in the porcine AI laboratories. The aim of the study conducted by Ribas-Maynou et al. was to evaluate the practical suitability of some of the most used methods. They tested eight methods, namely the direct measurement methods TUNEL, TUNEL with decondensation, the alkaline and neutral Comet assays, and chromomycin A3 (CMA3); and the indirect methods sperm chromatin structure assay (SCSA), and the alkaline and neutral sperm chromatin dispersion (SCD) assays. Based on the ability to measure the degree of chromatin damage, the study concluded that direct methods are more suitable than indirect methods. Finally, Pérez-Gómez et al. provided a timely and comprehensive review summarizing the events that take place during preimplantation embryo development in ungulates in order to identify genes regulating lineage differentiation. The review also discusses studies reporting altered lineage development in embryos produced *in vitro* and, finally, provides a general view of molecular markers of lineage differentiation. In this regard, they emphasize that transcriptomics is a powerful tool for discovering molecular markers of embryo quality.

Taken together, these papers demonstrate that omics are powerful tools to provide a more in depth understanding of reproductive events and to identify molecular markers to select best breeders or improve reproductive performance of livestock species.

## Author Contributions

All authors have contributed to the writing of this editorial and have approved it for publication.

## Funding

CM and IB were financially supported by the European Union's Horizon 2020 research and innovation program (grants H2020- MSCA-IF-2019-891663 and H2020- MSCA-IF-2019-891382).

## Conflict of Interest

The authors declare that the research was conducted in the absence of any commercial or financial relationships that could be construed as a potential conflict of interest.

## Publisher's Note

All claims expressed in this article are solely those of the authors and do not necessarily represent those of their affiliated organizations, or those of the publisher, the editors and the reviewers. Any product that may be evaluated in this article, or claim that may be made by its manufacturer, is not guaranteed or endorsed by the publisher.
